# Liver cancer segmentator: Metadata-guided confidence scoring for reliable segmentation of colorectal liver metastases in CT*

**DOI:** 10.1016/j.cmpb.2026.109233

**Published:** 2026-01-03

**Authors:** Mohammad Hamghalam, Jacob J. Peoples, Kaitlyn S.M. Kobayashi, Grace Park, Erin Kwak, E. Claire Bunker, Natalie Gangai, Mithat Gonen, Yun Shin Chun, HyunSeon Christine Kang, Richard K.G. Do, Amber L. Simpson

**Affiliations:** a School of Computing, Queen’s University, Kingston, ON, Canada; b Department of Epidemiology and Biostatistics, Memorial Sloan Kettering Cancer Center, New York, NY, USA; c Department of Surgical Oncology, The University of Texas MD Anderson Cancer Center, Houston, TX, USA; d Department of Electrical Engineering, Qa.C., Islamic Azad University, Qazvin, Iran; e Department of Abdominal Imaging, The University of Texas MD Anderson Cancer Center, Houston, TX, USA; f Department of Radiology, Memorial Sloan Kettering Cancer Center, New York, NY, USA; g Department of Biomedical and Molecular Sciences, Queen’s University, Kingston, ON, Canada

**Keywords:** Confidence-aware segmentation, Liver tumor segmentation, Colorectal liver metastases (CRLM), NnU-Net, Contrast-enhanced CT (CECT)

## Abstract

**Background and Objective:**

This study introduces the liver cancer segmentator (LCS), a deep learning model designed for automatic and robust segmentation of liver parenchyma and tumors in abdominal contrast-enhanced computed tomography images from patients with colorectal liver metastases. The primary aim was to enhance confidence scoring for more reliable clinical segmentation assessment.

**Methods:**

In this retrospective study, 446 abdominal contrast-enhanced computed tomography examinations were collected; 355 (80%) were used for training and 91 for testing. Data originated from routine clinical cases at two institutions, representing diverse disease stages and treatment settings. A state-of-the-art neural network segmentation framework was trained on these cases, with performance evaluated using the Dice score and the normalized surface distance. An iterative training process, supported by an integrated annotation workflow, was employed to refine the training set. The final model was applied to the 91 test examinations to assess the impact of tumor volume and slice thickness on confidence scoring. Reliability was quantified through pairwise Dice score for failure detection and the area under the risk coverage curve.

**Results:**

The LCS achieved a Dice score of 0.9707 (95% CI: 0.9663–0.9751) for liver parenchyma and 0.7695 (95% CI: 0.7166–0.8224) for tumors. Normalized surface distance values at a 3-millimeter tolerance were 0.9605 (95% CI: 0.9539–0.9671) for parenchyma and 0.8412 (95% CI: 0.7928–0.8896) for tumors. Confidence scoring analysis demonstrated strong correlations between tumor volume, slice thickness, and segmentation reliability, reducing the area under the risk coverage curve from 16.7 to 10.3.

**Conclusions:**

The LCS achieved high segmentation accuracy in patients with colorectal liver metastases. Incorporating tumor volume and slice thickness into the confidence scoring process improved failure detection, enhanced reliability, and provided valuable insights for refining clinical deployment of automated segmentation algorithms.

## 1. Introduction

Colorectal cancer is the second leading cause of cancer-related death worldwide [1]. Nearly half of these patients will develop liver metastases (colorectal liver metastases (CRLM)) with <10% surviving past 3 years [2,3]. Precise assessment of CRLM is vital for treatment decision-making including thermal percutaneous ablation, radiotherapy, surgical resection, arterial embolization, and systemic chemotherapy [4,5]. Abdominal contrast-enhanced CT (CECT) scans are central to this evaluation, providing detailed imaging for tumor burden assessment and staging [3,4].

### Clinical challenges.

The appearance of tumors in CT scans is affected by variation in (1) image acquisition and reconstruction protocol (contrast agent, contrast timing, resolution, reconstruction kernels, *etc*), (2) tumor biology (mucinous content, fibrosis, necrosis, treatment effects), and (3) underlying liver disease (steatosis, steatohepatitis, sinuisoidal obstruction syndrome) [6]. Manual segmentation of liver tumors is labor-intensive, poorly reproducible, subjective, and requires specialized expertise, making it especially challenging in cases with complex metastatic patterns [6]. Response evaluation criteria in solid tumors (RECIST) require the measurement of target lesions, but for each organ only up to two lesions are measured in their maximal axial diameters [7], which underrepresents the total tumor burden. In CRLM in particular, tumors are small and distributed throughout the liver, and are difficult to trend over time in treated patients. Fully automated liver tumor segmentation in CT images would bridge clinical gaps but remains a challenge [8].

### Technical challenges.

Recent advances in deep learning have revolutionized medical image segmentation [9–11] and detection [12]. Frameworks like nnU-Net achieve state-of-the-art performance by automatically optimizing architectures and hyperparameters for specific datasets [13]. Incorporating iterative annotation workflows, where human-refined contours are fed to the segmentation engine, has proven particularly effective for abdominal CT segmentation tasks [14]. Despite these advances, clinical deployment is hindered by variations in imaging protocols (e.g., slice thickness, contrast timing) and scanner heterogeneity [15,16]. Even top-performing models fail catastrophically when encountering out-of-distribution data, highlighting the need for reliable failure detection [17]. Existing methods for segmentation reliability emphasize uncertainty estimation [18–21], out-of-distribution detection [22], and quality regression [23], with the Area Under the Risk Coverage Curve (AURC) emerging as a critical evaluation metric [24]. However, these approaches often neglect key factors such as slice thickness and tumor size, despite their significant impact on segmentation performance. Subcentimeter tumors (<1 cm), for example, are particularly prone to segmentation errors due to partial volume effects and boundary ambiguity. Therefore, for eventual clinical translation, a robust liver tumor segmentation method should be able to handle tumors of all sizes, including small ones found in patients with CRLM, while also providing reliable failure detection. To address these challenges, we developed the liver cancer segmentator (LCS), a deep-learning system designed specifically for CRLM parenchyma and tumor segmentation in portal venous CT scans. In particular, small CRLMs are difficult to segment due to reduced contrast relative to the surrounding parenchyma, irregular lesion morphology, and heightened susceptibility to partial-volume effects [9].

Complementary advances in segmentation and clinical AI [25,26]—including a real-time segmentation framework [27], an XGBoost risk model for early tumor detection [28], attention-enhanced U-Net methods for lung nodule analysis [29], a hybrid FrCN–U-Net architecture for skin lesion segmentation [30], and volumetric/replicator networks for MRI brain tumor analysis [31]—offer additional methodological perspectives for model design and evaluation. Additionally, broader mathematical and reliability-oriented studies on graph-theoretic structures, fractional integro-differential formulations, reliability-based design optimization using differential–algebraic equations, and fuzzy-logic reliability allocation [32–38] provide conceptual grounding for robustness and risk quantification that can inform confidence-scoring strategies.

While previous benchmarks and nnU-Net based pipelines have established strong baselines for liver and tumor segmentation (e.g., LiTS [8]), they commonly evaluate on mixed hepatic disease cohorts and do not explicitly incorporate acquisition metadata into reliability estimates for clinical deployment. Recent surveys on uncertainty estimation [39] highlight that ensemble and Bayesian approaches improve failure detection but often ignore scan-level acquisition factors (slice thickness, reconstruction kernel) and lesion-level descriptors (tumor volume) that strongly influence segmentation reliability. Related liver tumor classification efforts [40,41] further demonstrate the growing interest in machine learning methods for hepatic disease analysis. Our work addresses these gaps by (i) training and evaluating on a multi-institutional CRLM-specific cohort that includes small, diffuse metastases typical of colorectal disease, and (ii) introducing a metadata-enhanced confidence score that combines ensemble pairwise agreement with slice-thickness and per-lesion volume weighting to prioritize cases for expert review. These additions reduce the AURC substantially on our held-out test set while maintaining high segmentation accuracy.

Segmentation reliability is crucial for ensuring clinician confidence. To address the uncertainties inherent in segmentation models, we developed a confidence scoring methodology. This method quantifies the reliability of each prediction and identifies cases requiring further review. By incorporating both model agreement [19] and domain-specific factors, our framework enhances the interpretability and robustness of CRLM segmentations, providing clinicians with a measure of prediction certainty. To assess the reliability of our confidence estimates, we evaluated the AURC [24], which quantifies the trade-off between risk and coverage, with lower values indicating better confidence calibration.

The proposed framework — referred to as the LCS — integrates segmentation and metadata-aware confidence scoring. The key contributions of our work are:

- The LCS, a deep learning model trained on 446 multi-institutional CT scans, achieving a Dice similarity coefficient (DSC) of 0.9707 (95% CI: 0.9663–0.9751) for liver parenchyma and 0.7695 (95% CI: 0.7166–0.8224) for tumors – comparable to radiologist performance.
- A metadata-enhanced confidence scoring framework incorporating tumor size thresholds (<1 cm) and slice thickness (>5 mm) into reliability estimates, reducing AURC from 16.7 to 10.3 – showing a clinician where the models fail.
- The public release of the segmentation model, confidence scoring framework, and source code to facilitate clinical translation.^1^

## 2. Materials and methods

**Fig. 1.**
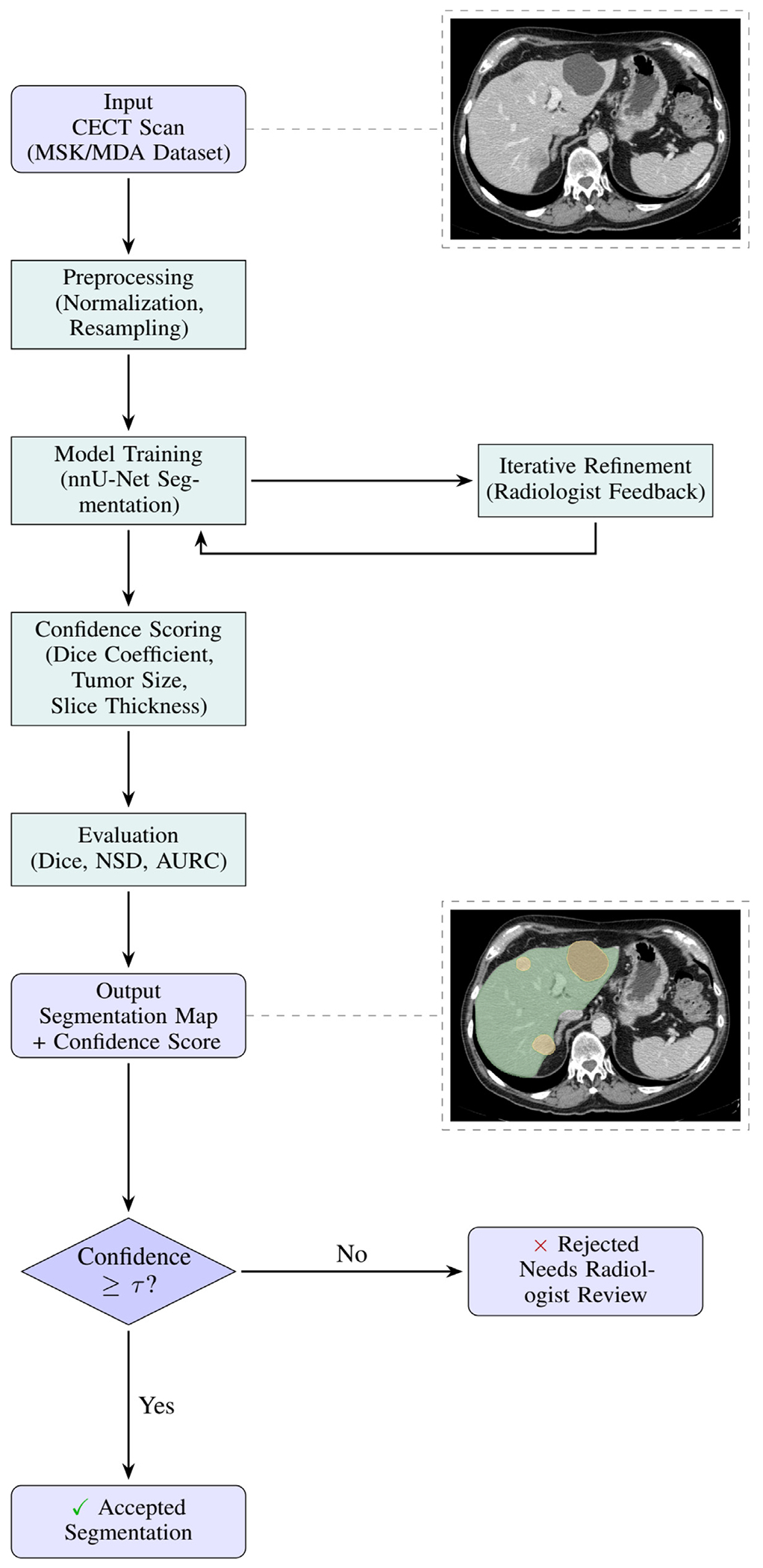
End-to-end LCS pipeline with confidence-based quality control. Input CT scans are preprocessed, segmented using nnU-Net, and evaluated for confidence. Segmentations meeting the confidence threshold (*τ*) are accepted (✓), while those below the threshold are rejected (×) and flagged for radiologist review. Example images illustrate the input CT scan and corresponding segmentation output.

The overall system, termed the LCS, consists of preprocessing, segmentation, iterative refinement, and metadata-guided confidence scoring. We developed a confidence-aware deep learning pipeline (Fig. 1) for automated CRLM segmentation in CT scans. The workflow consists of preprocessing, model training, iterative refinement of segmentation outputs, and confidence scoring to ensure robustness and interpretability. Our approach incorporates data from CRLM patients across different disease stages. A confidence-based quality control step evaluates segmentation reliability: predictions exceeding a predefined confidence threshold (*τ*) are accepted, while lower-confidence segmentations are flagged for radiologist review. This ensures that unreliable predictions are identified and mitigated before clinical application.

### 2.1. Datasets

This multi-institutional study analyzed 446 portal venous phase CT examinations from patients with CRLM. Scans were acquired from Memorial Sloan Kettering Cancer Center (MSK) and the University of Texas MD Anderson Cancer Center (MDA). The data set included publicly available ‘‘TCIA’’ data [42], retrospectively collected cohorts of patients at different cancer stages, and a prospectively collected cohort of patients at all stages. By incorporating patients at different stages and images acquired under a variety of conditions, we aim to improve diversity of imaging data and generalizability of resulting models.

The dataset was composed of four cohorts:

- **Resection cohort**: This cohort includes patients prior to liver resection of CRLM queried from liver resection databases. Images were acquired prior to hepatic resection. Eligibility for liver resection is determined based on size, number, location and distribution of tumors, as well as maintaining sufficient future liver remnant [3]. In general, liver tumors are confined to a single lobe.
- **Chemotherapy cohort**: This cohort includes patients who received chemotherapy treatment queried from medical oncology databases. The images were acquired before and during treatment, so treatment effects are present in the images, such as decreases in enhancement (becoming darker on a contrast CT scan) and development of calcifications (bright dots on the image).
- **All stages cohort**: Patients in this cohort were prospectively enrolled in an imaging trial at the time of routine imaging. Images of patients at all stages and all types of treatment were therefore included, representing the full variability of patients seen at both institutions.
- **TCIA cohort**: This is a public dataset collected from MSK. The cohort consists of patients undergoing resection, meeting the same criteria as those in the ‘‘Resection’’ cohort above.

**Fig. 2.**
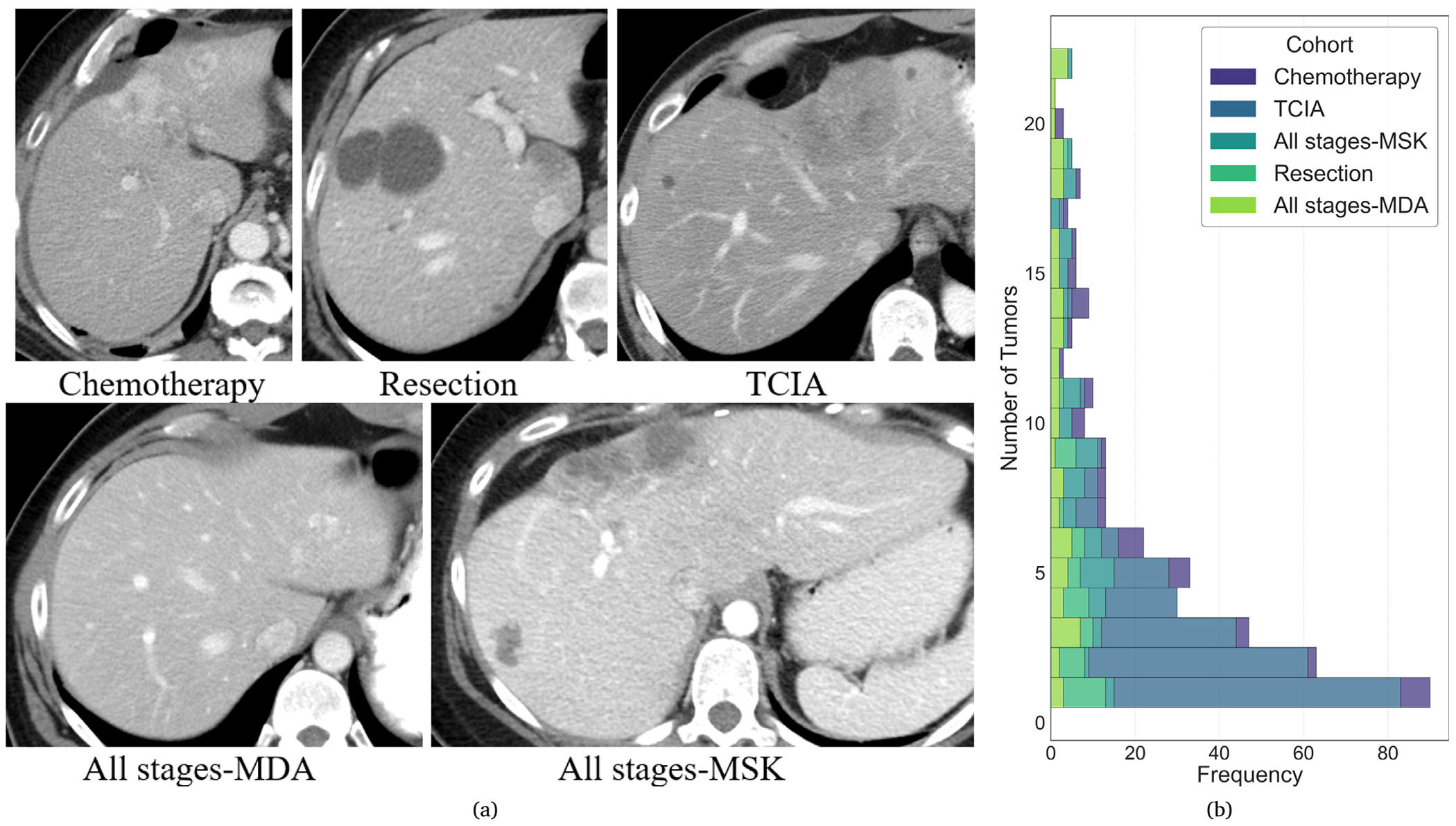
(a) presents representative contrast-enhanced CT samples from the five cohorts in our dataset — “Chemotherapy”, “Resection”, “TCIA”, “All stages-MDA’, and “All stages-MSK” — illustrating the challenges of CRLM segmentation. (b) shows the distribution of tumor counts across cases in the dataset.

Fig. 2(a) shows the challenges of CRLM segmentation in different stages and treatments of cancer. Fig. 2(b) shows the distribution of tumor counts across the patients in our study demonstrating the wide variability of the imaging data use for training and evaluation in our study.

**Fig. 3.**
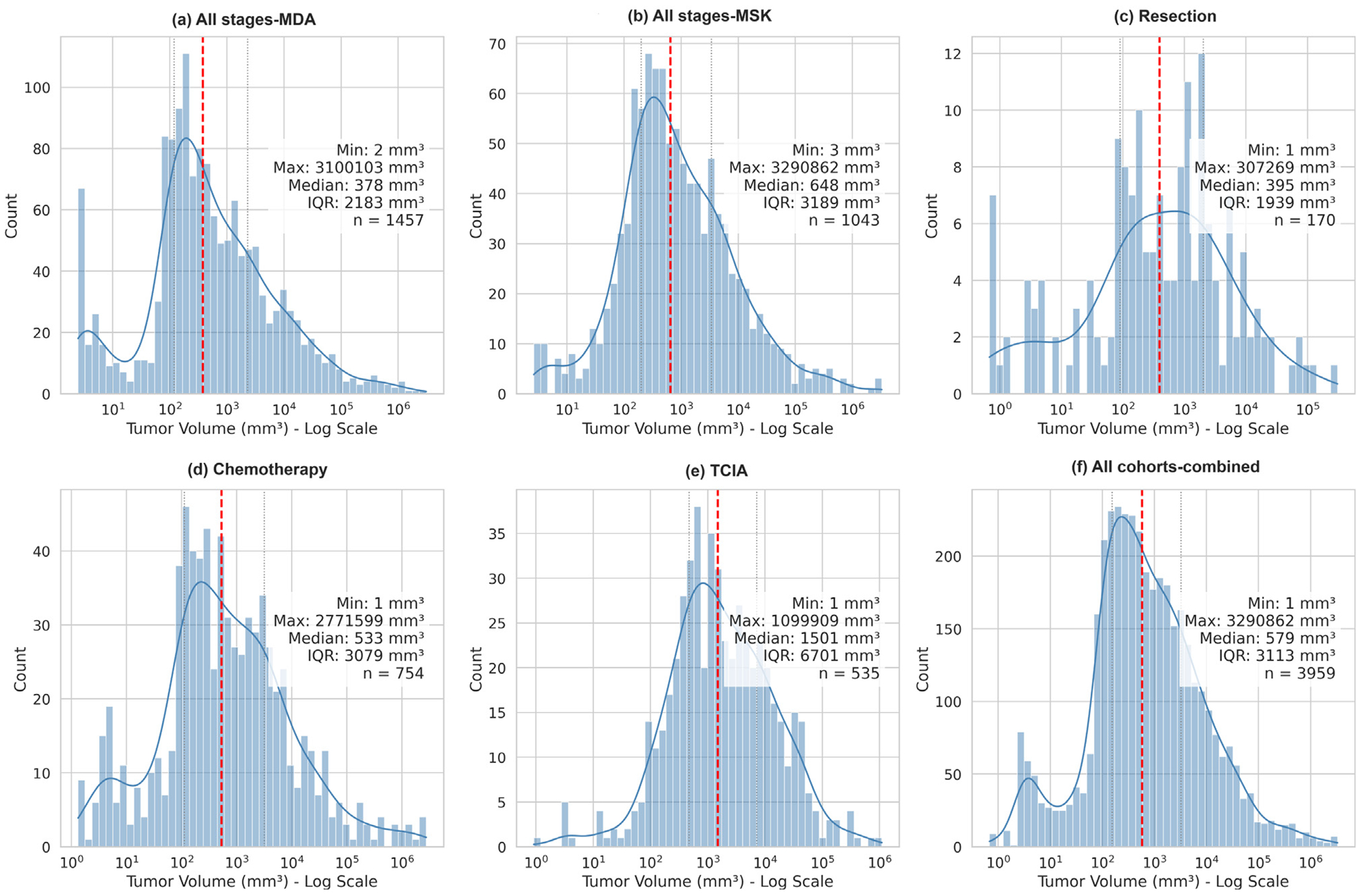
Comparative analysis of tumor volume distributions across experimental cohorts. (a–e) Cohort-specific histograms with kernel density estimation (KDE) showing tumor volume distributions (log_10_ scale), with red dashed lines indicating cohort medians. (f) Combined distribution from all cohorts demonstrating population-level trends. Sample sizes (*n*) and median values are annotated in each panel.

In the combined dataset (*n* = 3959 tumors) (Fig. 3(f)), the median tumor volume was 0.579 cm^3^ (equivalent sphere diameter ~10.4 mm) with an interquartile range (IQR) of 3.1 cm^3^ (equivalent sphere diameter ~18.2 mm). All cohorts demonstrated right-skewed distributions, with the MDA cohort showing the most pronounced skewness and kurtosis, indicative of a high concentration of smaller tumor volumes. These results underscore the importance of interpreting tumor volume distributions in a cohort-specific context, accounting for differences in imaging protocols.

CT series were randomly sampled across institutions to minimize selection bias. The dataset was further stratified by institution and divided into an 80% training/validation set (355 examinations) and a 20% independent test set (91 examinations). All ground truth tumor and liver segmentations were manually annotated using 3D Slicer software [43], following standardized protocols established by a panel of board-certified abdominal radiologists. Final segmentations were reviewed and approved by a senior radiologist with over 10 years of post-fellowship experience.

Tables 1 and 2 summarize the dataset characteristics. Table 1 provides tumor volume statistics across cohorts, including mean and median tumor volume, diameter, and average tumor counts. Table 2 compares key parameters between the training and test sets, with values reported as median along with their interquartile ranges (IQR). This includes measures of in-plane resolution, slice thickness, volume size, tumor counts, and both liver and tumor volumes, as well as the intensity of liver and tumor regions in Hounsfield Units (HU).

### 2.2. Imaging protocols

All abdominal CT scans were acquired during the portal venous phase following protocols aligned with institutional standard of care protocols. Imaging was conducted using multi-detector CT systems (predominantly GE Discovery, LightSpeed, Revolution series; GE Healthcare, Madison, WI, USA) with detector configurations spanning 4–128 rows, detector widths of 0.6–1.25 mm, and total collimation widths of 10–80 mm. A subset of MDA scans in the ‘‘Resection’’ and ‘‘Chemotherapy’’ cohorts was acquired using scanners from other vendors. Tube voltage was 100–140 kVp, and most commonly 120 kVp. Tube current was either fixed (MSK: mean 272–340 mA, range 119–755 mA; MDA: mean 283–326 mA, range 148–720 mA) or dynamically adjusted via GE smart mA tube current modulation with variable noise index (NI) and current range (NI: 8–20 for MSK and 9–33 for MDA; tube current 220–380 mA for MSK, 275–650 mA for MDA). Gantry rotation times were most commonly 0.7–0.8 s, with pitch 0.984, although a rotation time of 0.5 s and pitch factor of 0.516 was also common at MDA. Reconstruction utilized soft-tissue kernels across all studies. Section thicknesses varied from sub-millimeter (0.625 mm) to 7.5 mm, with contiguous slices. Adaptive statistical iterative reconstruction (ASiR) was applied in more recent scans, typically at a level of 20%.

### 2.3. Iterative training strategy

We employed a six-phase curriculum learning strategy for training the segmentation model. In each phase, additional cohorts of CRLM patients were introduced to the training set, progressively improving the model’s generalization capability. Starting with a smaller dataset in Phase 1, we incrementally incorporated new data from different institutions and disease stages. The specific number of samples introduced at each training phase is detailed in Table 3. These steps form the training workflow of the LCS.

To further improve segmentation accuracy, the model was iteratively refined using expert corrections. After each training phase, model outputs were reviewed by an abdominal radiologist (>10 years of post-fellowship experience). Errors were manually corrected, and the refined annotations were reintroduced into the training set, allowing the model to progressively learn from challenging cases. This iterative feedback loop, depicted in Fig. 1, ensured continuous performance improvements across diverse imaging protocols and clinical conditions.

### 2.4. Segmentation model

The segmentation framework is based on a 3D Residual Encoder U-Net (ResEncUNet) built on nnU-Net [13,44] and corresponds to the model trained during Phase 5, which yielded the best performance. The final 3D architecture comprises seven encoder–decoder stages with feature depths [32, 64, 128, 256, 320, 320, 320]. All convolutional layers use 3 × 3 × 3 kernels, instance normalization, and LeakyReLU activations (*α* = 0.01). Downsampling is implemented via strided convolutions, with stride patterns chosen to preserve higher axial resolution in deeper stages. Input patches of 96 × 256 × 256 voxels were used to balance memory constraints and field-of-view requirements. This model configuration serves as the core segmentation engine of the LCS.

Preprocessing and planning followed nnU-Net conventions with adaptations for anisotropic CT data. Images were read with SimpleITK and normalized using the nnU-Net CT scheme (foreground 0.5–99.5th percentile ≈ 9–208 HU; mean ≈ 111 HU; SD ≈ 34.9 HU), followed by z-score standardization. Resampling used nnU-Net’s default routines (cubic interpolation for image data; linear/nearest for segmentations and probability maps) to a target spacing of [1.5, 0.8066, 0.8066] mm to preserve axial detail across scans with slice thickness ranging from 0.8–7.5 mm. For completeness, the planner also produced a 2D configuration (patch size 512 × 512, batch size 35) and a 3D full-resolution configuration (patch size 96 × 256 × 256, batch size 2); the latter was employed as our final model.

#### Data splits and leakage prevention.

All dataset partitioning was performed at the examination (patient) level to prevent any image from the same patient being present in both training and test sets. The multi-institutional data were stratified by cohort and split into an 80% training/validation set (355 examinations) and a 20% independent held-out test set (91 examinations), as reported in Table 3. Preprocessing (resampling, intensity normalization) and any hyperparameter selection were performed strictly within training/validation folds; final model evaluation used only the held-out test set. For robustness assessment we also conducted a 5-fold cross-validation on the combined MSK+MDA institutional cohorts (reported in Section 3.5). Ensemble models (N = 5) were trained using distinct training splits; ensemble averaging and pairwise agreement were computed only after independent model training. These procedures minimize the risk of data leakage and ensure the test set reflects an unseen sample distribution.

### 2.5. Confidence scoring methodology

As part of the LCS, we introduce a metadata-enhanced confidence scoring module designed to quantify prediction reliability. To quantify the reliability of each segmentation prediction, we developed a confidence scoring methodology that integrates both model agreement and domain-specific factors. This framework provides a measure of prediction certainty and allows for the identification of low-confidence predictions requiring further review. Below, we describe the key components of our confidence scoring methodology, including pairwise agreement among ensemble models, domain-specific adjustments, and the evaluation of confidence scores using the AURC.

#### 2.5.1. Pairwise agreement and confidence score

To detect potential failures, we computed pairwise Dice similarity per case, taking the mean Dice coefficient across all unique pairs of model outputs. This calculation used the full segmentation mask for each case. For parenchyma and tumor, the same procedure was applied while restricting the Dice computation to the respective voxel regions. The baseline confidence score was derived from pairwise agreement between ensemble models [19].

For *N* = 5 models, we computed the DSC for each model pair as:

(1) $$\mathrm{DSC}\left(f_{i},f_{j}\right)=\frac{2\left|S_{f_{i}}\cap S_{f_{j}}\right|}{\left|S_{f_{i}}\right|+\left|S_{f_{j}}\right|},$$

where *S_f_i__* and *S_f_j__* are segmentation masks from models *f_i_* and *f_j_*. The ensemble confidence score was then calculated as:

(2) κpairwise=1(N2)∑i=1N∑j=i+1NDSCfi,fj.

#### 2.5.2. Confidence adjustments

The baseline confidence score was adjusted using two domain-specific factors:

**Slice Thickness Weighting:** Scans with *z*_spacing_ > 5.0 mm received reduced confidence due to thick-slice artifacts:

(3) $$\alpha_{\text{thickness}}=\left\{\begin{array}{ll} 1.0, & z_{\text{spacing}}\leq 5.0\;\mathrm{mm},\\ 5.0/z_{\text{spacing}}, & \text{otherwise}. \end{array}\right.$$

**Tumor Volume Weighting:** Small tumors (*V*_tumor_ ≤ *V*_lower_bound_) were penalized using linear interpolation between 0.5 and 1.0:

(4) $$\alpha_{\text{tumor}}=\left\{\begin{array}{ll} 0.5, & V_{\text{tumor}}\leq V_{\text{lower\_bound}},\\ 1.0, & V_{\text{tumor}}\geq V_{\text{upper\_bound}},\\ \frac{V_{\text{tumor}}-V_{\text{lower\_bound}}}{V_{\text{upper\_bound}}-V_{\text{lower\_bound}}}, & \text{otherwise}. \end{array}\right.$$

The final confidence score combined these components:

(5) $$\kappa_{\text{final},i}=\kappa_{\text{pairwise},i}\times \alpha_{\text{thickness},i}\times \alpha_{\text{tumor},i}.$$

##### Implementation details and parameter choices.

All experiments used an ensemble of *N* = 5 models and the pairwise agreement baseline defined in Eq. (2). We applied two domain-specific multiplicative adjustments to produce a final confidence score per prediction (Eq. (5)).

###### Slice thickness (*α-thickness*).

**Fig. 4.**
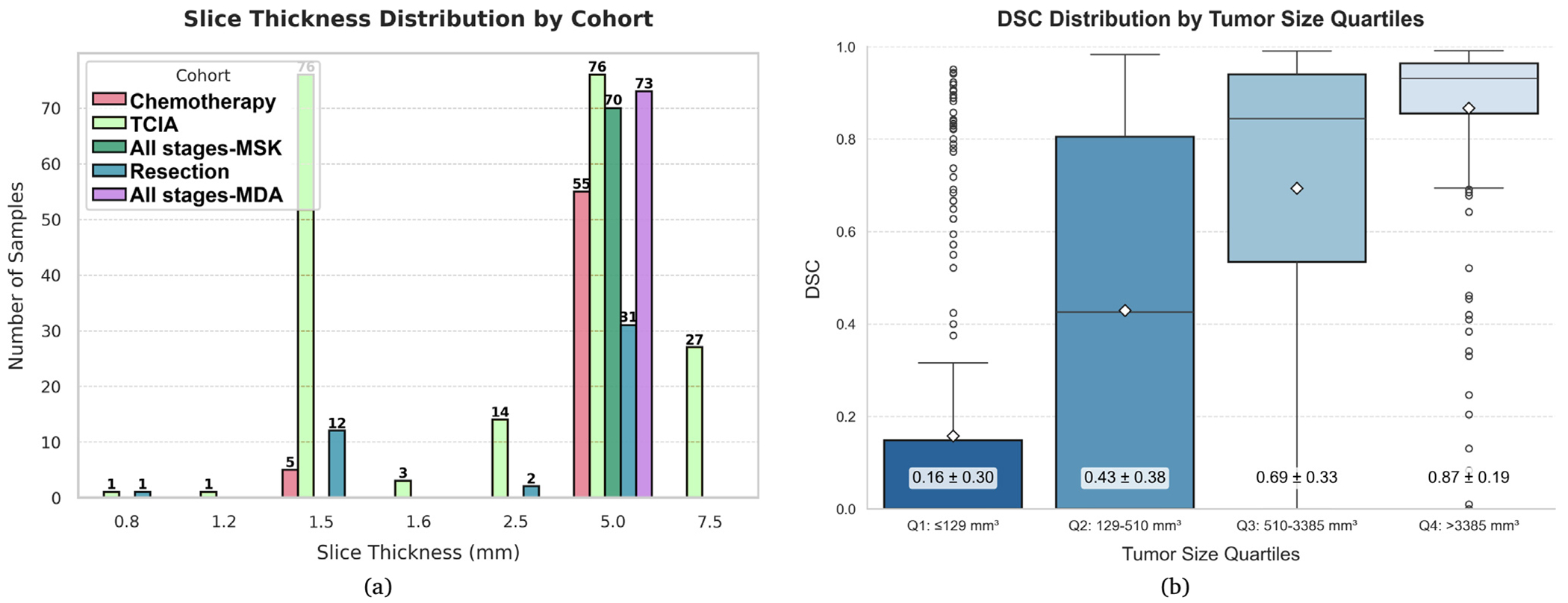
(a) Distribution of slice thickness across the dataset, illustrating variability in imaging protocols (range: 0.8–7.5 mm). (b) Dice score distribution by tumor size quartiles, highlighting segmentation challenges in smaller tumors.

We empirically selected a z-spacing threshold of 5.0 mm. Scans with *z_spacing* ≤ 5.0 mm keep full weight (*α*_thickness_ = 1.0). For thicker scans we applied the inverse linear scaling *α*_thickness_ = 5.0/*z-spacing* (Eq. (3)), which reduces confidence smoothly as slice thickness increases. This choice reflects the observed degradation in Dice for thicker reconstructions (Fig. 4(a)).

###### Tumor volume (*α-tumor*).

For tumor-level weighting we penalize very small lesions using the linear scheme in Eq. (4). In the experiments reported here we set *V*_lower_ = 0.51 cm^3^ (the 1st quartile cut-off in the combined dataset; cf. Fig. 4(b)) and *V*_upper_ = 2.0 cm^3^ (a pragmatic upper bound beyond which no penalty is applied). Thus lesions with *V*_tumor_ ≤ 0.51 cm^3^ receive *α*_tumor_ = 0.5, lesions with *V*_tumor_ ≥ 2.0 cm^3^ receive *α*_tumor_ = 1.0, and intermediate volumes are interpolated linearly. We chose these bounds to (i) penalize subcentimeter/sub-Q1 lesions strongly (which show high Dice variance) and (ii) avoid penalizing larger lesions whose segmentation was robust in our data (Fig. 4(b)).

###### Case/scan aggregation rule.

For scans with multiple tumors we computed *α*_tumor_ based on the *average tumor volume* across all lesions in that scan. Using the mean lesion size provides a balanced per-scan weighting that reflects the overall lesion burden without allowing a single very small lesion to disproportionately dominate the confidence score.

#### 2.5.3. AURC evaluation

To assess the reliability of our confidence estimates, we evaluated the AURC [24]. AURC quantifies the trade-off between risk and coverage, where lower values indicate better confidence calibration.

For each test case *x_i_*, we defined segmentation risk as:

(6) $$r_{i}=1-\mathrm{DSC}\left(y_{i},y_{\mathrm{gt}}\right),$$

where *y_i_* is the predicted segmentation and *y*_gt_ the ground truth. The risk-coverage curve was computed as:

(7) $$R_{s}(\tau )=\frac{\sum_{i=1}^{N}r_{i}\cdot I\left(\kappa_{\text{final},i}\geq \tau \right)}{\sum_{i=1}^{N}I\left(\kappa_{\text{final},i}\geq \tau \right)},$$

(8) $$C(\tau )=\frac{1}{N}\sum_{i=1}^{N}I\left(\kappa_{\text{final},i}\geq \tau \right),$$

where I is an indicator function.

AURC was computed following the approach in [45], adapted for segmentation tasks. The impact of metadata-based confidence adjustments on AURC is analyzed and reported.

## 3. Results

### 3.1. Tumor volume and equivalent diameter distributions across cohorts

The impact of metadata-based confidence adjustments on AURC is shown in Table 3. A comparative analysis of tumor volume distributions across experimental cohorts is illustrated in Fig. 3. Tumor volumes are reported in cm^3^, and equivalent sphere diameters (calculated from the volume assuming a spherical shape) are provided in parentheses for reference. The “All stages-MSK” cohort (Fig. 3(b)) comprised patients with more advanced, unresectable disease, contributing to its larger median tumor volumes compared to the MDA cohort (Fig. 3(a)), which included a more diverse CRLM population.

The All stages-MDA cohort (*n* = 1457 tumors) exhibited the lowest median tumor volume (0.378 cm^3^, equivalent sphere diameter ~8.9 mm), while the MSK cohort had higher median volumes (0.648 cm^3^, equivalent sphere diameter ~10.7 mm). Significant heterogeneity was observed across the cohorts, reflecting differences in patient populations and clinical contexts. The “Chemotherapy-MSK” cohort (Fig. 3(d)) (*n* = 754 tumors) had patients with unresectable disease and the highest tumor burden, with the largest tumor volume (representing a confluence of lesions) reaching 2771 cm^3^ (equivalent sphere diameter ~163.5 mm).

The “MSK resection” cohort (Fig. 3(c)), with a smaller number of tumors (*n* = 170), had a median tumor volume of 0.395 cm^3^ (equivalent sphere diameter ~9.1 mm), while the “TCIA-MSK” cohort (Fig. 3(e)) demonstrated the widest tumor volume range, from 1 mm^3^ (equivalent sphere diameter ~1.2 mm) to 1099 cm^3^(equivalent sphere diameter ~128.0 mm), with a median of 1.5 cm^3^ (equivalent sphere diameter ~14.2 mm).

Additionally, the dataset exhibited heterogeneous imaging parameters, with slice thicknesses in the test set ranging from 0.8 mm to 7.5 mm (Fig. 4(a)).

### 3.2. Impact of tumor size on segmentation performance

To explicitly link size distribution to model performance, we examined segmentation quality across tumor-size strata (Fig. 4(b)). In the combined dataset (*n* = 3959 tumors; median volume 0.579 cm^3^, IQR 3.1 cm^3^), lesions in the smallest quartile (≤0.51 cm^3^) exhibited substantially lower and more variable Dice scores compared with larger quartiles (Fig. 4(b)). This decrease in performance for small lesions is consistent with partial-volume effects, reduced lesion-to-parenchyma contrast and boundary ambiguity — factors that are exacerbated in scans with thicker reconstructions (slice thickness up to 7.5 mm; Fig. 4(a)). Quantitative quartile-level DSC statistics are shown in Fig. 4(b); a brief discussion of mitigation strategies is given in the Discussion.

### 3.3. Iterative training performance

We employed a six-phase iterative training strategy, progressively incorporating additional cohorts and refining model predictions using expert corrections (see Table 3). Model performance improved steadily across phases as more data and expert feedback were added. For liver parenchyma segmentation, the DSC increased from 0.9618 ± 0.0444 in Phase 1 to 0.9718 ± 0.0191 in Phase 6. For tumor segmentation, the DSC increased from 0.7106 ± 0.2815 in Phase 1 to 0.7584 ± 0.2590 in Phase 5. Although tumor performance showed greater variability, the overall upward trend highlights the value of iterative refinement for more challenging structures. Overall, these results demonstrate that our multi-phase training strategy reduced the need for manual corrections and minimized radiologists’ involvement.

For all six training phases, we initially used nnU-Net v1. Based on the tumor DSC, Phase 5 showed the strongest performance for tumor segmentation. When the improved nnU-Net v2 became available, we applied its ResEncUNet architecture [44] specifically to Phase 5 to obtain better results while conserving time and computational resources. Although nnU-Net v2 typically outperforms v1 across all phases, we limited its use to the best-performing phase to avoid re-running all previous experiments.

Table 3 reports the performance achieved with nnU-Net v1 across all phases. In contrast, the Phase 5 ResEncUNet model achieved a liver parenchyma DSC of 0.9707 (95% CI: 0.9663–0.9751). For tumor segmentation, it achieved a DSC of 0.7695 (95% CI: 0.7166–0.8224), representing an improvement over the nnU-Net v1 tumor DSC of 0.7584.

The iterative training approach, combined with the final ResEncUNet model in Phase 5, leveraged NVIDIA A100 GPUs (40 GB) to efficiently process the large datasets and integrate the latest modifications to the model architecture and annotations.

### 3.4. Segmentation performance across phases

**Fig. 5.**
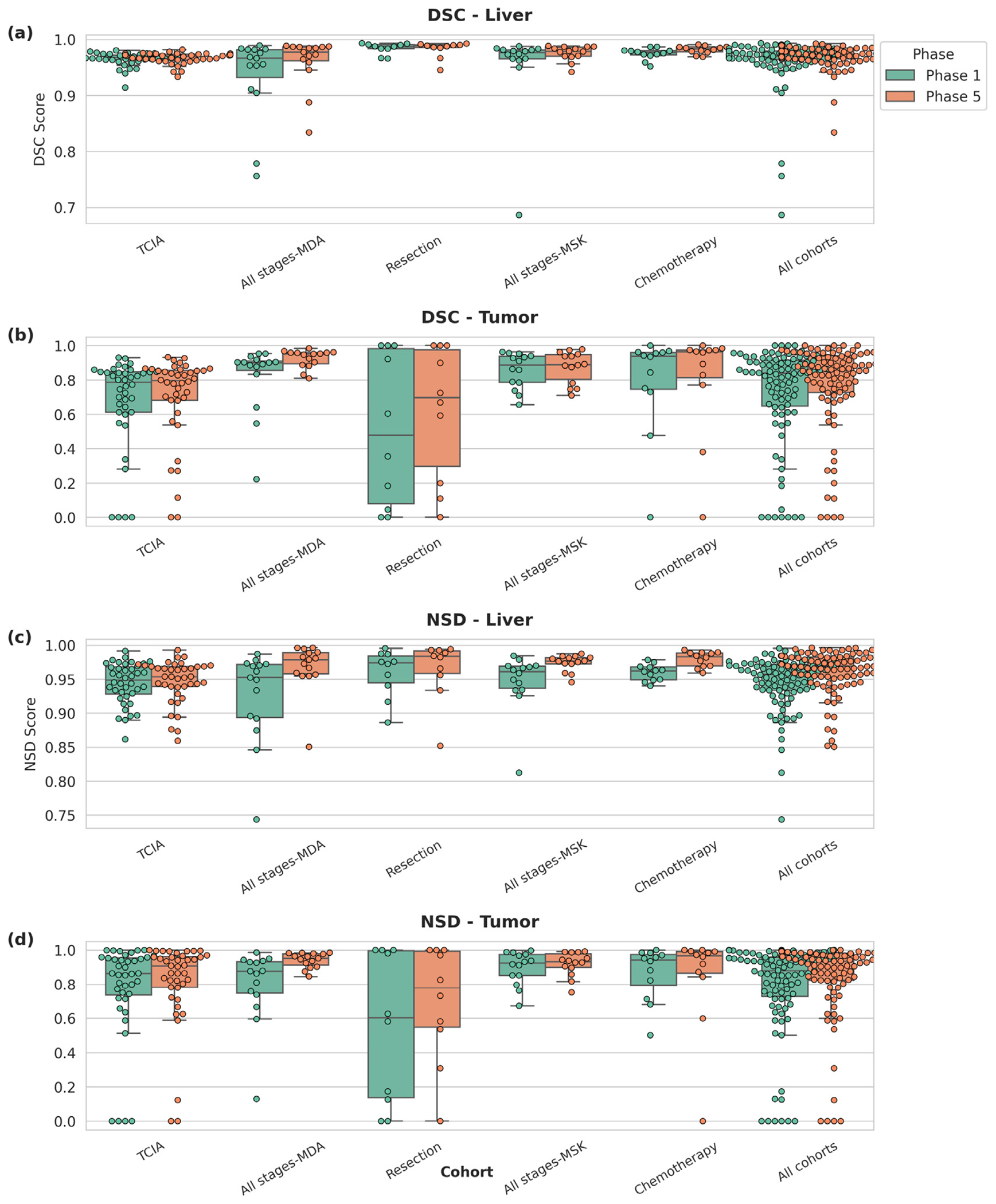
Comparison of segmentation performance (DSC and NSD with 3 mm tolerance) between Phase 1 (green) and Phase 5 (red) for liver and tumor across various cohorts. The boxplots display the distribution of scores for each phase, while the swarmplots represent individual data points for enhanced visual insight. The *x*-axis corresponds to the different cohorts, and the *y*-axis represents the segmentation scores. Phase 1 corresponds to the early training stage, while Phase 5 represents the final model after integrating additional data batches. Cohorts include: “Resection”, “Chemotherapy”, “All stages-MDA”, “All stages-MSK”, “TCIA”, and “All” cohorts.

The boxplots and swarmplots in Fig. 5 present a comparison of segmentation performance in Phase 1 and Phase 5 using the DSC and the Normalized Surface Distance (NSD, 3 mm tolerance; a metric quantifying the percentage of surface points with inter-surface distances ≤3, mm) for both liver and tumor structures.

For liver segmentation, the model demonstrated consistent DSC improvement across cohorts, with the “All” cohort increasing from 0.9618 ± 0.0446 in Phase 1 to 0.9707 ± 0.0213 in Phase 5. Similar improvements were observed in other cohorts, such as “All stages-MDA” (from 0.9361 ± 0.0732 to 0.9610 ± 0.0438) and “All stages-MSK” (from 0.9537 ± 0.0776 to 0.9756 ± 0.0134), suggesting that liver segmentation accuracy benefited from the incorporation of additional training data.

Tumor segmentation showed more modest improvements compared to liver segmentation. The “All” cohort displayed an increase in tumor DSC from 0.7233 ± 0.2831 in Phase 1 to 0.7695 ± 0.2573 in Phase 5. The “All stages-MDA” cohort, however, exhibited a more significant improvement, with the tumor DSC rising from 0.8175 ± 0.2007 to 0.9247 ± 0.0521, suggesting that the model refined its tumor segmentation capabilities in this cohort. For NSD, liver segmentation also showed a clear improvement from Phase 1 to Phase 5, with the “All stages-MDA” cohort increasing from 0.9264 ± 0.0663 to 0.9680 ± 0.0359. In contrast, tumor segmentation results were more variable, with the “Resection” cohort showing a smaller increase in NSD from 0.5485 ± 0.4380 in Phase 1 to 0.6949 ± 0.3406 in Phase 5.

Overall, the results indicate that the model’s performance improved over the training phases, particularly in liver segmentation. Tumor segmentation, while showing measurable improvement, exhibited greater variability across cohorts and phases. This variability is expected given that tumor segmentation is inherently more challenging than liver segmentation, partly due to inter-radiologist variability in assessing tumor burden. In fact, the model nearly achieved human-level accuracy for tumor segmentation, suggesting that simply adding more samples may yield diminishing returns. Further improvements in tumor segmentation may require alternative strategies beyond increasing the dataset size.

### 3.5. Impact of tumor size on segmentation accuracy

Fig. 4(b) illustrates a positive correlation between tumor size and segmentation accuracy. Mean DSC increased by 435% from the smallest tumor quartile (Q1) to the largest (Q4). Standard deviation showed an inverse correlation with tumor size, decreasing from 0.30 in Q1 to 0.19 in Q4. While larger tumors (Q3–Q4) achieved clinically relevant performance (DSC > 0.65), smaller tumors (Q1–Q2) exhibited greater variability, particularly in Q2, where the standard deviation (0.38) was close to the mean value (0.43). These findings underscore the need for specialized strategies to improve segmentation of lesions smaller than 0.51 cm^3^.

### 3.6. Cross-validation and model complexity

To complement the held-out test evaluation, we performed a 5-fold cross-validation on the combined MSK and MDA institutional cohorts and report both overall stability and cohort-level behavior. Over the cross-validation folds the liver segmentation achieved a mean DSC of 0.9707 ± 0.0012 and tumor segmentation a mean DSC of 0.7703 ± 0.0120, indicating low variance across splits and stable aggregate performance.

**Fig. 6.**
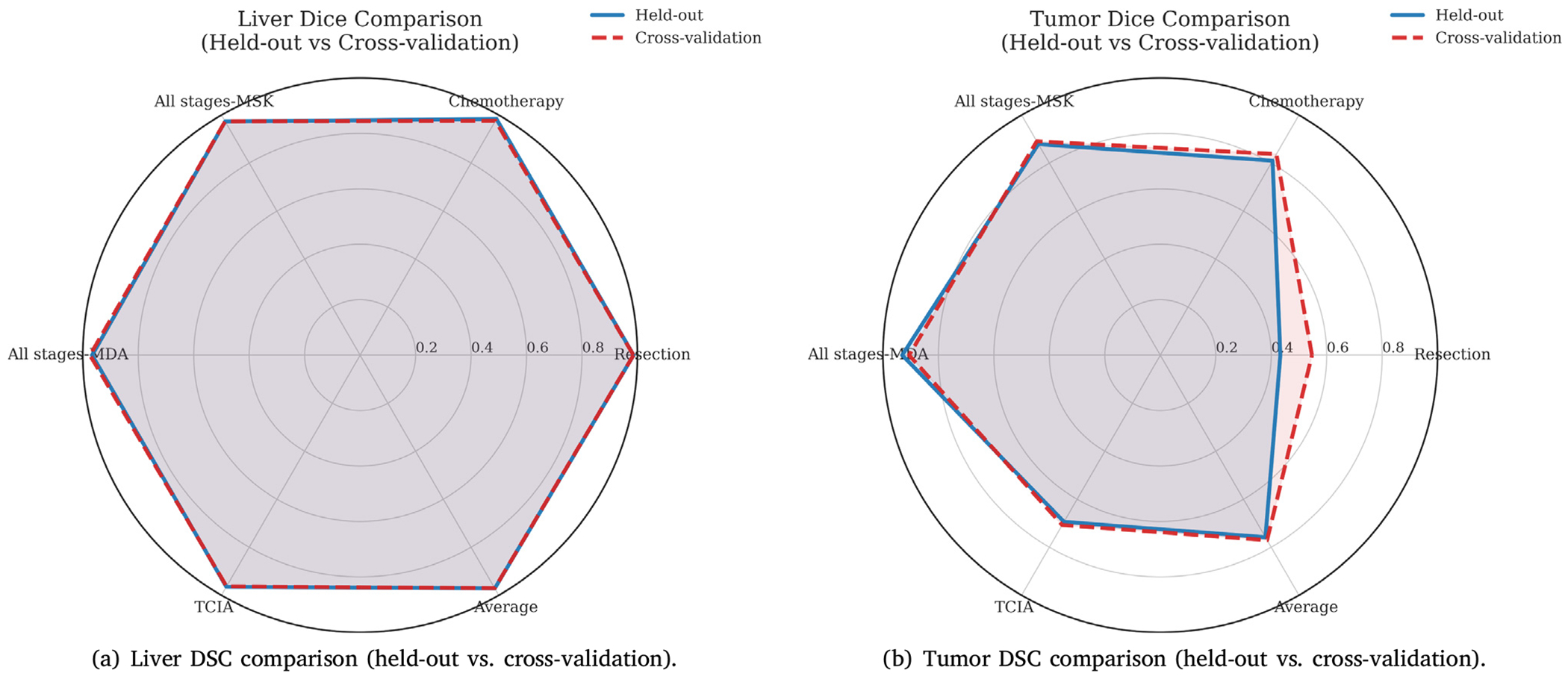
Spider (radar) plots comparing the mean DSC performance for held-out testing and 5-fold cross-validation across all cohorts. Panel (a) shows liver segmentation performance, which remains consistently high across cohorts with close agreement between held-out and cross-validation scores. Panel (b) shows tumor segmentation performance, which presents greater cohort-dependent variability, reflecting differences in lesion size distributions and imaging characteristics.

To inspect cohort-specific agreement between validation and the independent test set, we aggregated per-cohort mean DSC from cross-validation and compared these to held-out test DSC using radar (spider) plots (Figs. 6(a) and 6(b)). The Liver radar (Fig. 6(a)) shows consistently high parenchyma DSC across cohorts (held-out mean ≈ 0.971; cross-validation mean ≈ 0.971), indicating that liver segmentation is robust to cohort- and site-level differences. By contrast, the Tumor radar (Fig. 6(b)) reveals larger between-cohort variability: for example, the Resection cohort has a substantially lower held-out tumor DSC (0.433) than its cross-validation average (0.547), whereas the All stages-MDA cohort attains very high tumor DSC on both held-out (0.931) and cross-validation (0.906) evaluations. Overall, cross-validation means closely track held-out performance (overall tumor: held-out ≈ 0.758 vs. CV ≈ 0.770), but cohort-level discrepancies highlight the effect of differing lesion distributions and imaging conditions and motivate the metadata-aware confidence scoring and site-specific calibration described in Section 2.5.

We also quantified model complexity and runtime characteristics for practical deployment. The final 3D ResEncUNet contains 141,073,790 trainable parameters and requires roughly 7277.78 GMac per forward pass on a full-resolution input patch. Average inference for a complete test volume on our evaluation GPU was approximately 2 min. These measurements provide a realistic estimate of computational requirements for prospective clinical use and can guide resource planning for deployment.

### 3.7. Improvement of failure detection through confidence scoring adjustments

**Fig. 7.**
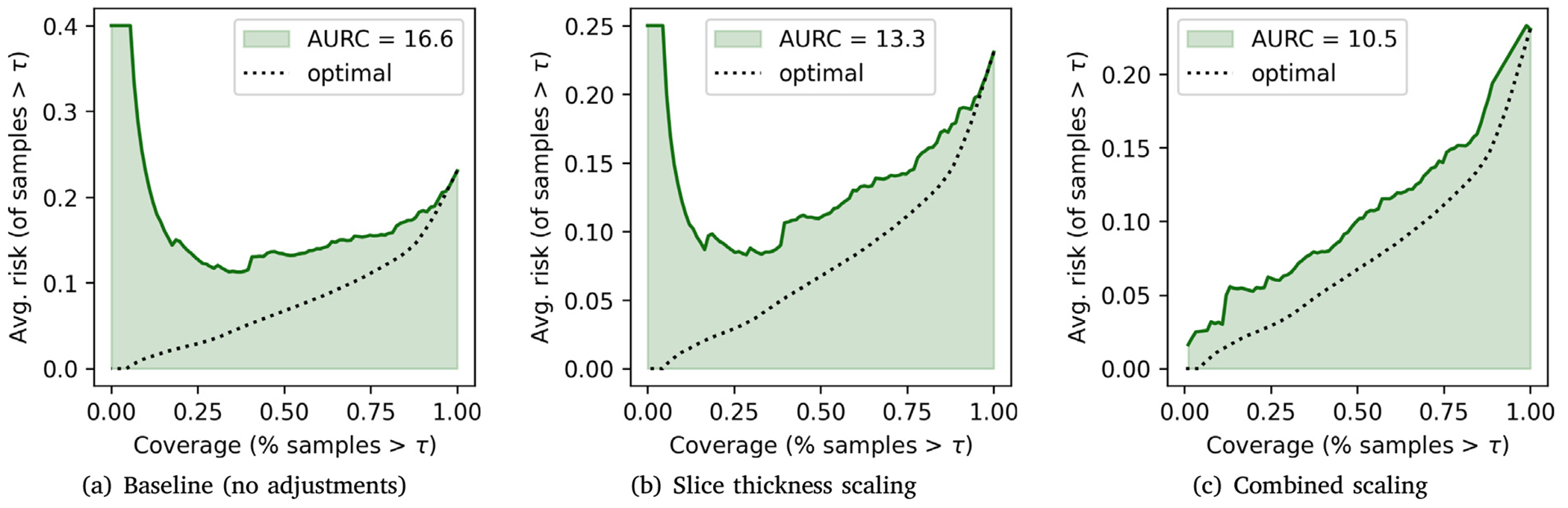
Comparison of confidence scoring strategies using the AURC. Lower AURC indicates better failure detection. (a) Baseline: No adjustments (AURC = 16.6). (b) Slice thickness scaling: Penalizes *z*_spacing_ > 5.0 mm (AURC = 13.3). (c) Combined scaling: Adds tumor size thresholds (AURC = 10.5).

Incorporating slice thickness and tumor size into confidence scoring significantly improved failure detection performance, reducing the AURC from 16.6 (baseline) to 13.3 (slice thickness scaling) and ultimately to 10.5 (combined scaling) (Fig. 7). This result aligns with the theoretical optimal AURC framework proposed by [24], where the ideal confidence score is defined as:

(9) $$\kappa_{\text{optimal}}=-\text{risk}=-(1-\text{DSC}).$$

Although the optimal AURC, which is theoretically near 0 (since a perfect segmentation with DSC = 1 would yield zero risk), serves as a lower bound, in practice we focus on the relative improvements in AURC across different weighting strategies rather than reporting the absolute optimal value, which is rarely achieved due to inherent segmentation uncertainties.

The AURC quantifies uncertainty estimation quality by integrating segmentation risk (1 – DSC) across confidence thresholds. Unlike traditional metrics such as Pearson (*ρ*) or Spearman (*r_s_*) correlations — which measure rank associations — AURC provides a clinically interpretable benchmark where lower values directly reflect improved failure detection [24].

Table 4 highlights the model performance improvements between Phase 1 (single-cohort training) and Phase 5 (multi-cohort training) across various CRLM patient cohorts. Table 4 shows the AURC, Spearman, and Pearson correlation coefficients, which are used to evaluate segmentation reliability and confidence. Lower AURC values reflect improved failure detection, with the combined tumor volume and slice thickness weighting strategy yielding the lowest AURC values, particularly in the “All stages-MDA” cohort.

The results demonstrate a substantial improvement from Phase 1 to Phase 5 in most cohorts, with AURC reductions such as from 8.39 to 4.06 in the “All stages-MDA” cohort and from 42.70 to 22.53 in the “Resection-MSK” cohort. These findings underscore the efficacy of multi-cohort training and metadata-driven confidence adjustments in enhancing segmentation robustness across diverse patient populations and imaging protocols.

## 4. Discussion

### 4.1. Key findings

Our proposed LCS demonstrated high segmentation accuracy. The model achieved a DSC of 0.9713 for liver parenchyma and 0.7584 for liver tumors, resulting in an average combined liver+tumor DSC of 0.8649. For context, a recent benchmarking study (“nnU-Net Revisited”) [46] reports average combined liver+tumor DSCs of 0.8234 for CNN-based methods, 0.7910 for Transformer-based methods, and 0.8040 for Mamba-based methods on the LiTS dataset [8]. Our combined DSC of 0.8649 therefore represents an absolute improvement of +0.0415 (≈4.15 percentage points) over the CNN-based benchmark, +0.0739 (≈7.39 percentage points) over the Transformer-based benchmark, and +0.0609 (≈6.09 percentage points) over the Mamba-based benchmark.

We emphasize that direct comparisons should be interpreted with caution. The benchmark results were obtained on LiTS, whereas our results are based on a larger, multi-institutional dataset with different imaging characteristics and a broader range of clinical cases; these differences can affect absolute performance. Nevertheless, the explicit gains reported above strengthen the claim that our iterative training and multi-institutional data contributed to improved and more robust segmentation performance. Finally, the integration of an iterative training framework — progressively refining the training set with expert-guided corrections — reduced the need for manual corrections and helped minimize radiologists’ intervention in finalizing segmentations, supporting clinical applicability.

### 4.2. Metadata-enhanced confidence scoring

A key contribution of this study is the development of a metadata-enhanced confidence scoring framework that incorporates tumor size and slice thickness. Unlike traditional uncertainty-based methods, which often fail to account for clinically relevant imaging variabilities, our approach reduced the AURC from 16.6 (baseline) to 10.5 (combined scaling) by penalizing predictions on small tumors and thick-slice acquisitions. This reduction underscores the value of metadata-driven reliability estimation, particularly for clinical deployment where segmentation errors could significantly impact treatment decisions. The confidence scoring framework also demonstrated strong correlations between tumor volume, slice thickness, and segmentation reliability. For a focused discussion of the particular challenges posed by small lesions (which show markedly lower and more variable DSC; see Fig. 4(b)), and our mitigation strategies, see the Limitations and Future Work subsection below.

### 4.3. Clinical implications

The ability to flag low-confidence segmentations using metadata-derived confidence scores provides a mechanism for targeted expert review, thereby enabling radiologists to focus on cases that require manual intervention. By prioritizing these cases, the framework has the potential to reduce radiologists’ workload while maintaining high segmentation accuracy. In our study, the observed reduction in manual corrections suggests that confidence-aware segmentation models could streamline radiological workflows and enhance overall efficiency. Moreover, the public release of our trained models and source code supports reproducibility and encourages broader adoption of the model in both clinical and research settings.

In addition to these workflow benefits, it is important to recognize the clinical limitations and appropriate use-cases of the proposed model. Although the system achieves high liver accuracy and strong tumor performance for larger lesions, segmentation of subcentimeter tumors remains challenging, as reflected by the lower DSC observed in the smallest quartile (Fig. 4(b)). Performance is also affected by imaging quality, particularly thicker reconstructions (up to 7.5 mm), which introduce partial-volume artifacts. For these reasons, the model is best used as an assistive tool that automatically accepts high-confidence cases while directing radiologists’ attention to low-confidence or small-lesion cases flagged by the metadata-enhanced confidence score. Prospective validation and site-specific calibration are recommended before deployment, and thin-slice acquisitions should be preferred when accurate assessment of small lesions is clinically required.

Clinically, our LCS can function as an assistive, triage-capable tool that automatically accepts high-confidence scans while flagging low-confidence cases for radiologist review, potentially reducing manual correction burden and improving measurement consistency (liver DSC = 0.9713, tumor DSC = 0.7584, combined = 0.8649; AURC reduced 16.6 → 10.5). However, subcentimeter lesions and thick-slice reconstructions remain important limitations — the model should therefore be used with human-in-the-loop review, site-specific calibration, and preference for thin-slice imaging when small-lesion assessment is critical.

### 4.4. Limitations and future work

Despite the strong segmentation performance, several limitations remain. Small tumors pose particular challenges for automated segmentation in portal-venous CT. In our data the lesion size distribution is heavily right-skewed and a large fraction of lesions are subcentimeter (per-lesion median ≈ 10.4 mm; several cohorts have median diameters *<*10–11 mm), and the smallest lesions in the TCIA subset approach 1 mm^3^ in volume. Importantly, lesions in the smallest quartile (≤0.51 cm^3^) exhibit substantially lower and more variable DSC (Fig. 4(b)), which we attribute to partial-volume effects, reduced lesion-to-parenchyma contrast, and boundary ambiguity. These issues are exacerbated by thicker reconstructions (slice thickness up to 7.5 mm). To mitigate these effects we (i) resampled to preserve axial detail during preprocessing, (ii) incorporated slice-thickness and tumor-volume weighting into our confidence score to flag likely failures, and (iii) used iterative human-in-the-loop corrections and an ensemble strategy to improve robustness. For clinical deployment we recommend — where feasible — use of thinner reconstructions or targeted high-resolution scanning for small-lesion workups, lesion-aware loss weighting or focal-loss formulations, and targeted augmentation or oversampling of subcentimeter lesions during training to reduce variability in the smallest quartiles.

While the proposed metadata-enhanced confidence score improves failure detection (AURC reduced from 16.6 to 10.5; Fig. 6), it has limitations that warrant explicit discussion. First, the score relies on a small set of heuristic adjustments (slice-thickness scaling and tumor-volume interpolation) whose thresholds (e.g., *Z*-spacing = 5.0 mm, *V*_lower_ = 0.51 cm^3^, *V*_upper_ = 2.0 cm^3^) were selected empirically; these values are reported in Section 2.5 and may require site-specific recalibration. Second, our per-scan aggregation rule (mean lesion volume) provides a balanced estimate of lesion burden but can under-emphasize a single clinically important tiny lesion in scans with multiple larger lesions; alternative aggregation choices (minimum or median) offer different safety/performance trade-offs and should be considered based on clinical priorities. Third, ensemble pairwise agreement may be biased if the ensemble shares common failure modes or lacks architectural diversity, and the current score does not explicitly quantify epistemic uncertainty for out-of-distribution inputs (e.g., unusual reconstruction kernels or vendor-specific artifacts). Finally, annotation bias and class imbalance (a preponderance of small lesions) can influence both model predictions and the estimated confidence.

To reduce these risks we recommend the following next steps: carry out sensitivity analyses for threshold and aggregation choices, evaluate complementary uncertainty estimators (e.g., Bayesian/MC-dropout or ensembles with greater architectural diversity), perform prospective site-level calibration and validation, and retain a human-in-the-loop review step for low-confidence predictions. Future work will also explore advanced augmentation, super-resolution or targeted acquisition protocols to improve small-lesion performance and the integration of principled uncertainty quantification to better capture out-of-distribution risk. Prospective multi-center evaluation and clinical workflow integration remain prerequisites for safe deployment.

## 5. Conclusion

We presented an iterative, expert-in-the-loop training strategy combined with a metadata-enhanced confidence scoring framework that together materially improve automated segmentation of liver parenchyma and CRLM. On our multi-institutional dataset the system achieved strong segmentation accuracy (liver DSC ≈ 0.97; tumor DSC ≈ 0.76; combined DSC ≈ 0.865), improved reliability in failure detection (AURC reduced from 16.6 to 10.5 with combined scaling), and reduced the need for manual corrections during label curation—demonstrating both technical performance gains and practical workflow benefits.

These quantitative gains translate into two complementary clinical advantages: (1) reliable automatic acceptance of high-confidence cases, which can streamline routine volumetric reporting and reduce radiologist time spent on trivial edits; and (2) targeted triage of low-confidence cases to expert review, which concentrates human effort where it matters most. At the same time, important limitations temper immediate deployment: segmentation performance degrades for subcentimeter lesions (smallest quartile ≤ 0.51 cm^3^), thicker reconstructions increase partial-volume errors, the confidence score depends on empirically chosen thresholds that may require site-specific recalibration, and ensemble agreement can be susceptible to common failure modes and annotation bias.

To translate these results into safe clinical practice we recommend the following next steps: prospective, multi-center validation and site-level calibration of confidence thresholds; integration of principled uncertainty estimators (e.g., Bayesian/MC-dropout or more architecturally diverse ensembles); targeted strategies to improve small-lesion performance (high-resolution acquisitions, super-resolution, lesion-aware loss weighting, and focused augmentation/oversampling); evaluation of alternative per-scan aggregation rules (minimum/median) to prioritize clinically important tiny lesions; and user-interface and workflow studies to quantify radiologist time-savings and error modes in real-world use. Finally, continued public release of models, code, and evaluation scripts will facilitate external replication, safety auditing, and regulatory review—necessary steps before widespread clinical deployment.

In summary, our approach advances the reliability and clinical readiness of deep learning–based liver and tumor segmentation by combining improved accuracy with metadata-aware reliability estimation, while also identifying clear technical and validation milestones that must be met prior to routine clinical adoption.

## Figures and Tables

**Table 1:** Overview of tumor characteristics across cohorts. Tumor volume and diameter statistics reflect heterogeneity in lesion size and burden.

Cohort	Institution	Mean tumor (Std) volume (cm^3^)	Median tumor volume (cm^3^)	Largest tumor diameter (mm)	Mean tumor count	# Scans
Resection	MSK	21.8 (54.4)	3.7	26.1	4.4	46
Chemotherapy	MSK	415.5 (715.4)	69.2	84.6	13.7	60
All stages	MDA	317.4 (524.6)	99.9	87.0	20.0	73
All stages	MSK	304.6 (678.1)	40.5	73.1	14.9	70
TCIA	MSK	36.0 (106.0)	7.7	34.0	2.7	197

**Table 2:** Comparison of training and test sets. Values are reported as median (IQR). Abbreviations: HU = Hounsfield Units.

Parameter	Train (n = 355) Median (IQR)	Test (n = 91) Median (IQR)
In-plane resolution (mm)	0.81 (0.75–0.90)	0.83 (0.78–0.90)
Slice thickness (mm)	5.00 (2.50–5.00)	5.00 (5.00–5.00)
Volume size (voxels)	512 × 512 × 53 (47–87.5)	512 × 512 × 53 (47–63)
Number of tumors	4 (2–10)	4 (1–10)
Tumor volume (cm^3^)	24.9 (5.9–146.4)	23.6 (6.4–121.2)
Liver volume (cm^3^)	1524.3 (1242.1–1830.8)	1541.9 (1310.6–1814.1)
Liver intensity (HU)	113.0 (99.0–127.9)	109.2 (99.9–122.6)
Tumor intensity (HU)	79.1 (65.0–91.7)	73.9 (64.6–89.0)

**Table 3:** Data distribution and segmentation performance (DSC) across training phases. Cohorts are stratified by institution (MSK/MDA) and clinical context. Test samples reflect 20% held-out per cohort. Mean and standard deviation (std) of liver and tumor DSC values are reported per phase.

Cohort (Institution)	Phase 1	Phase 2	Phase 3	Phase 4	Phase 5	Phase 6	Test
TCIA (MSK)	157	157	157	157	157	157	40
All stages (MSK)	0	29	29	29	29	56	14
All stages (MDA)	0	0	32	32	32	58	15
Chemotherapy (MSK)	0	0	0	48	48	48	12
Resection (MSK)	0	0	0	0	36	36	10
Total	157	187	219	267	303	356	91

Liver DSC	0.9618 ± 0.0444	0.9675 ± 0.0293	0.9692 ± 0.0277	0.9705 ± 0.0230	0.9713 ± 0.0202	**0.9718 ± 0.0191**	–

Tumor DSC	0.7106 ± 0.2815	0.7403 ± 0.2778	0.7404 ± 0.2827	0.7495 ± 0.2738	**0.7584 ± 0.2590**	0.7486 ± 0.2688	–

**Table 4:** Comparison of model performance between Phase 1 (Single-Cohort Training) and Phase 5 (Multi-Cohort Training). The table shows AURC (Area Under the Risk Coverage Curve) values, as well as Spearman and Pearson correlations for each cohort and phase. The weighting strategies (slice thickness and tumor volume) were applied to assess model confidence and failure detection across cohorts from different institutions (MSK and MDA). Lower AURC values indicate better failure detection.

Cohort	Phase	Weighting	Metrics		
			AURC	Spearman	Pearson
	Phase 1	Slice thickness + Tumor volume	8.39	−0.79	−0.91
	Phase 1	Slice thickness	8.39	−0.79	−0.92
All stages-MDA	Phase 1	None	8.39	−0.79	−0.92
	Phase 5	Slice thickness + Tumor volume	4.06	−0.90	−0.92
	Phase 5	Slice thickness	4.06	−0.90	−0.92
	Phase 5	None	4.06	−0.90	−0.92

	Phase 1	Slice thickness + Tumor volume	8.50	−0.73	−0.57
	Phase 1	Slice thickness	8.50	−0.73	−0.57
All stages-MSK	Phase 1	None	8.50	−0.73	−0.57
	Phase 5	Slice thickness + Tumor volume	6.38	−0.87	−0.78
	Phase 5	Slice thickness	6.38	−0.87	−0.78
	Phase 5	None	6.38	−0.87	−0.78

	Phase 1	Slice thickness + Tumor volume	42.70	−0.41	−0.56
	Phase 1	Slice thickness	31.35	−0.57	−0.60
Resection-MSK	Phase 1	None	31.35	−0.57	−0.60
	Phase 5	Slice thickness + Tumor volume	22.53	−0.40	−0.57
	Phase 5	Slice thickness	26.69	−0.51	−0.52
	Phase 5	None	26.69	−0.51	−0.52

	Phase 1	Slice thickness + Tumor volume	8.46	−0.60	−0.43
	Phase 1	Slice thickness	6.38	−0.93	−0.57
Chemotherapy-MSK	Phase 1	None	6.38	−0.93	−0.57
	Phase 5	Slice thickness + Tumor volume	5.05	−0.63	−0.92
	Phase 5	Slice thickness	4.08	−0.94	−0.96
	Phase 5	None	4.08	−0.94	−0.96

	Phase 1	Slice thickness + Tumor volume	19.09	−0.68	−0.85
	Phase 1	Slice thickness	20.53	−0.56	−0.69
TCIA-MSK	Phase 1	None	27.57	−0.48	−0.59
	Phase 5	Slice thickness + Tumor volume	17.50	−0.68	−0.79
	Phase 5	Slice thickness	19.61	−0.53	−0.63
	Phase 5	None	26.42	−0.49	−0.59

## Footnotes

1 https://github.com/hamghalam/liverCancerSegmentator.

## References

[1] Bray Freddie, Laversanne Mathieu, Sung Hyuna, Ferlay Jacques, Siegel Rebecca L, Soerjomataram Isabelle, Jemal Ahmedin, Global cancer statistics 2022: GLOBOCAN estimates of incidence and mortality worldwide for 36 cancers in 185 countries, CA: Cancer J. Clin 74 (3) (2024) 229–263. 38572751 10.3322/caac.21834

[2] Steele G Jr., Ravikumar TS, Resection of hepatic metastases from colorectal cancer. Biologic perspective, Ann. Surg 210 (2) (1989) 127–138. 2667471 10.1097/00000658-198908000-00001 PMC1357818

[3] Engstrand Jenny, Nilsson Henrik, Strömberg Cecilia, Jonas Eduard, Freedman Jacob, Colorectal cancer liver metastases – a population-based study on incidence, diagnosis, and treatment patterns, Cancer Imaging 18 (1) (2018) 32, 10.1186/s40644-018-0161-9. 29334918 PMC5769309

[4] Cervantes A, Adam R, Roselló S, Arnold D, Normanno N, Taïeb J, Seligmann J, De Baere T, Osterlund P, Yoshino T, Martinelli E, ESMO Guidelines Committee, clinicalguidelines@esmo.org, Metastatic colorectal cancer: ESMO clinical practice guideline for diagnosis, treatment and follow-up, Ann. Oncol 34 (1) (2023) 10–32. 36307056 10.1016/j.annonc.2022.10.003

[5] Martin Jack, Petrillo Angelica, Smyth Elizabeth C, Shaida Nadeem, Khwaja Samir, Cheow HK, Duckworth Adam, Heister Paula, Praseedom Raaj, Jah Asif, Balakrishnan Anita, Harper Simon, Liau Siong, Kosmoliaptsis Vasilis, Huguet Emmanuel, Colorectal liver metastases: Current management and future perspectives, World J. Clin. Oncol 11 (10) (2020) 761–808. 10.5306/wjco.v11.i10.761 PMC7643190 33200074

[6] Moghbel Mehrdad, Mashohor Syamsiah, Mahmud Rozi, Saripan M Iqbal Bin, Review of liver segmentation and computer assisted detection/diagnosis methods in computed tomography, Artif. Intell. Rev 50 (4) (2018) 497–537.

[7] Eisenhauer EA, Therasse P, Bogaerts J, Schwartz LH, Sargent D, Ford R, Dancey J, Arbuck S, Gwyther S, Mooney M, Rubinstein L, Shankar L, Dodd L, Kaplan R, Lacombe D, Verweij J, New response evaluation criteria in solid tumours: revised RECIST guideline (version 1.1), Eur. J. Cancer 45 (2) (2009) 228–247. 19097774 10.1016/j.ejca.2008.10.026

[8] Bilic Patrick, Christ Patrick, Bran Li Hongwei, Vorontsov Eugene, Ben-Cohen Avi, Kaissis Georgios, Szeskin Adi, Jacobs Colin, Humpire Mamani Gabriel Efrain, Chartrand Gabriel, Lohöfer Fabian, Holch Julian Walter, Sommer Wieland, Hofmann Felix, Hostettler Alexandre, Naama Lev-Cohain Michal Drozdzal, Amitai Michal Marianne, Vivanti Refael, Sosna Jacob, Ezhov Ivan, Sekuboyina Anjany, Navarro Fernando, Kofler Florian, Paetzold Johannes C, Shit Suprosanna, Hu Xiaobin, Lipková Jana, Rempfler Markus, Piraud Marie, Kirschke Jan, Wiestler Benedikt, Zhang Zhiheng, Hülsemeyer Christian, Beetz Marcel, Ettlinger Florian, Antonelli Michela, Bae Woong, Bellver Míriam, Bi Lei, Chen Hao, Chlebus Grzegorz, Erik B Dam Qi Dou, Fu Chi-Wing, Georgescu Bogdan, Giró-I-Nieto Xavier, Gruen Felix, Han Xu, Heng Pheng-Ann, Hesser Jürgen, Hendrik Moltz Jan, Igel Christian, Isensee Fabian, Paul Jäger Fucang Jia, Kaluva Krishna Chaitanya, Khened Mahendra, Kim Ildoo, Kim Jae-Hun, Kim Sungwoong, Kohl Simon, Konopczynski Tomasz, Kori Avinash, Krishnamurthi Ganapathy, Li Fan, Li Hongchao, Li Junbo, Li Xiaomeng, Lowengrub John, Ma Jun, Maier-Hein Klaus, Maninis Kevis-Kokitsi, Meine Hans, Merhof Dorit, Pai Akshay, Perslev Mathias, Petersen Jens, Pont-Tuset Jordi, Qi Jin, Qi Xiaojuan, Rippel Oliver, Roth Karsten, Sarasua Ignacio, Schenk Andrea, Shen Zengming, Torres Jordi, Wachinger Christian, Wang Chunliang, Weninger Leon, Wu Jianrong, Xu Daguang, Yang Xiaoping, Chun-Ho Yu Simon, Yuan Yading, Yue Miao, Zhang Liping, Cardoso Jorge, Bakas Spyridon, Braren Rickmer, Heinemann Volker, Pal Christopher, Tang An, Kadoury Samuel, Soler Luc, Ginneken Bram van, Greenspan Hayit, Joskowicz Leo, Menze Bjoern, The liver tumor segmentation benchmark (LiTS), Med. Image Anal 84 (102680) (2023). 10.1016/j.media.2022.102680 PMC10631490 36481607

[9] Hamghalam Mohammad, Do Richard K.G., Simpson Amber L., Attention-based CT scan interpolation for lesion segmentation of colorectal liver metastases, in: Medical Imaging 2023: Biomedical Applications in Molecular, Structural, and Functional Imaging, Vol. 12468, SPIE, 2023, pp. 186–193.

[10] Mojtahedi Ramtin, Hamghalam Mohammad, Do Richard KG, Simpson Amber L, Towards optimal patch size in vision transformers for tumor segmentation, in: International Workshop on Multiscale Multimodal Medical Imaging, Springer, 2022, pp. 110–120.

[11] Hamghalam Mohammad, Wang Tianfu, Qin Jing, Lei Baiying, Transforming intensity distribution of brain lesions via conditional gans for segmentation, in: 2020 IEEE 17th International Symposium on Biomedical Imaging, ISBI, IEEE, 2020, pp. 1–4.

[12] Hamghalam Mohammad, Moreland Robert, Gomez David, Simpson Amber, Ming Lin Hui, Jandaghi Ali Babaei, Tafur Monica, Vlachou Paraskevi A, Wu Matthew, Brassil Michael, , Machine learning detection and characterization of splenic injuries on abdominal computed tomography, Can. Assoc. Radiol. J 75 (3) (2024) 534–541. 38189316 10.1177/08465371231221052

[13] Isensee Fabian, Jaeger Paul F, Kohl Simon AA, Petersen Jens, Maier-Hein Klaus H, nnU-net: a self-configuring method for deep learning-based biomedical image segmentation, Nature Methods 18 (2) (2021) 203–211. 33288961 10.1038/s41592-020-01008-z

[14] Wasserthal Jakob, Breit Hanns-Christian, Meyer Manfred T, Pradella Maurice, Hinck Daniel, Sauter Alexander W, Heye Tobias, Boll Daniel T, Cyriac Joshy, Yang Shan, , TotalSegmentator: robust segmentation of 104 anatomic structures in CT images, Radiol.: Artif. Intell 5 (5) (2023). 10.1148/ryai.230024 PMC10546353 37795137

[15] AlBadawy Ehab A., Ashirbani Saha, Mazurowski Maciej A., Deep learning for segmentation of brain tumors: Impact of cross-institutional training and testing, Med. Phys 45 (3) (2018) 1150–1158. 29356028 10.1002/mp.12752

[16] Zech John R, Badgeley Marcus A, Liu Manway, Costa Anthony B, Titano Joseph J, Oermann Eric Karl, Variable generalization performance of a deep learning model to detect pneumonia in chest radiographs: a cross-sectional study, PLoS Med. 15 (11) (2018) e1002683. 30399157 10.1371/journal.pmed.1002683 PMC6219764

[17] Mehrtash Alireza, Wells William M, Tempany Clare M, Purang Abolmaesumi, Tina Kapur, Confidence calibration and predictive uncertainty estimation for deep medical image segmentation, IEEE Trans. Med. Imaging 39 (12) (2020) 3868–3878. 32746129 10.1109/TMI.2020.3006437 PMC7704933

[18] González Camila, Gotkowski Karol, Fuchs Moritz, Bucher Andreas, Dadras Armin, Fischbach Ricarda, Jasmin Kaltenborn Isabel, Mukhopadhyay Anirban, Distancebased detection of out-of-distribution silent failures for covid-19 lung lesion segmentation, Med. Image Anal 82 (2022) 102596. 36084564 10.1016/j.media.2022.102596 PMC9400372

[19] Roy Abhijit Guha, Conjeti Sailesh, Navab Nassir, Wachinger Christian, Alzheimer’s Disease Neuroimaging Initiative, , Bayesian QuickNAT: Model uncertainty in deep whole-brain segmentation for structure-wise quality control, NeuroImage 195 (2019) 11–22. 30926511 10.1016/j.neuroimage.2019.03.042

[20] Jungo Alain, Balsiger Fabian, Reyes Mauricio, Analyzing the quality and challenges of uncertainty estimations for brain tumor segmentation, Front. Neurosci 14 (2020) 282. 32322186 10.3389/fnins.2020.00282 PMC7156850

[21] Ng Matthew, Guo Fumin, Biswas Labonny, Petersen Steffen E, Piechnik Stefan K, Neubauer Stefan, Wright Graham, Estimating uncertainty in neural networks for cardiac MRI segmentation: a benchmark study, IEEE Trans. Biomed. Eng 70 (6) (2022) 1955–1966. 10.1109/TBME.2022.3232730 37015623

[22] Valindria Vanya V, Lavdas Ioannis, Bai Wenjia, Kamnitsas Konstantinos, Aboagye Eric O, Rockall Andrea G, Rueckert Daniel, Glocker Ben, Reverse classification accuracy: predicting segmentation performance in the absence of ground truth, IEEE Trans. Med. Imaging 36 (8) (2017) 1597–1606. 28436849 10.1109/TMI.2017.2665165

[23] Li Kang, Yu Lequan, Heng Pheng-Ann, Towards reliable cardiac image segmentation: Assessing image-level and pixel-level segmentation quality via self-reflective references, Med. Image Anal 78 (2022) 102426. 35367712 10.1016/j.media.2022.102426

[24] Zenk Maximilian, Zimmerer David, Isensee Fabian, Traub Jeremias, Norajitra Tobias, Jäger Paul F, Maier-Hein Klaus, Comparative benchmarking of failure detection methods in medical image segmentation: unveiling the role of confidence aggregation, Med. Image Anal 101 (2025) 103392. 39657400 10.1016/j.media.2024.103392

[25] Kujur Anima, Raza Zahid, Khan Arfat Ahmad, Wechtaisong Chitapong, Data complexity based evaluation of the model dependence of brain MRI images for classification of brain tumor and Alzheimer’s disease, IEEE Access 10 (2022) 112117–112133.

[26] Soleymanifard Mostafa, Hamghalam Mohammad, Segmentation of whole tumor using localized active contour and trained neural network in boundaries, in: 2019 5th Conference on Knowledge Based Engineering and Innovation, KBEI, IEEE, 2019, pp. 739–744.

[27] Shin Jungpil, Revolutionizing medical imaging with artificial intelligence real-time segmentation for enhanced diagnostics, EDRAAK 2024 (2024) 18–25.

[28] The role of artificial intelligence in early tumor detection: An XGBoost risk assessment model for Egyptian patients, Mesop. J. Artif. Intell. Healthc 2025 (2025) 85–92, 10.58496/MJAIH/2025/008, URL https://mesopotamian.press/journals/index.php/MJAIH/article/view/794.

[29] Aung Thin Myat Moe, Khan Arfat Ahmad, Enhanced U-net with attention mechanisms for improved feature representation in lung nodule segmentation, Curr. Med. Imaging 21 (1) (2025) E15734056386382. 10.2174/0115734056386382250902064757 PMC13223473 40947694

[30] Thapar Puneet, Rakhra Manik, Prashar Deepak, Mrsic Leo, Khan Arfat Ahmad, Kadry Seifedine, Skin cancer segmentation and classification by implementing a hybrid FrCN-(U-NeT) technique with machine learning, PLoS One 20 (6) (2025) e0322659. 40455780 10.1371/journal.pone.0322659 PMC12129148

[31] Rastogi Deependra, Johri Prashant, Donelli Massimo, Kadry Seifedine, Khan Arfat Ahmad, Espa Giuseppe, Feraco Paola, Kim Jungeun, Deep learning-integrated MRI brain tumor analysis: feature extraction, segmentation, and survival prediction using replicator and volumetric networks, Sci. Rep 15 (1) (2025) 1437. 39789043 10.1038/s41598-024-84386-0 PMC11718254

[32] Eze C, Imam T, Balarabe AM, O. Olaiya O, Graph-theoretic characterizations of Quasi-idempotents in full order-preserving transformation semigroup, Babylon. J. Math 2025 (2025) 88–91, 10.58496/BJM/2025/009, URL https://mesopotamian.press/journals/index.php/BJM/article/view/809.

[33] Mohammed Zaid A., Damak Mondher, Fadhel Fadhel S., Altahainah Hadeel Sameer, Existence and uniqueness theorem of multi-dimensional integro-differential equations with fractional differointegrations, Babylon. J. Math 2025 (2025) 44–49, 10.58496/BJM/2025/006, URL https://mesopotamian.press/journals/index.php/BJM/article/view/799.

[34] Abed Saad Abbas, Ghassan Mona, Latef Shaimaa Qais, Hind S. Hassan, Reliability-based design optimization using differential-algebraic equations, Iraqi J. Comput. Sci. Math 6 (3) (2025).

[35] Abed Saad Abbas, Khalil Zena H., Reliability allocation in complex systems using Fuzzy logic modules, Babylon. J. Math 2023 (2023) 78–83, 10.58496/BJM/2023/015, URL https://mesopotamian.press/journals/index.php/BJM/article/view/494.

[36] Soleimany Saeed, Hamghalam Mohammad, A novel random-valued impulse noise detector based on MLP neural network classifier, in: 2017 Artificial Intelligence and Robotics, IRANOPEN, 2017, pp. 165–169, 10.1109/RIOS.2017.7956461.

[37] Khan Arfat Ahmad, Driss Maha, Boulila Wadii, Sampedro Gabriel Avelino, Abbas Sidra, Wechtaisong Chitapong, Privacy preserved and decentralized smartphone recommendation system, IEEE Trans. Consum. Electron 70 (1) (2023) 4617–4624.

[38] Hamghalam Mohammad, Mirzakuchaki Sattar, Mohammad Ali Akhaee, Robust image watermarking using dihedral angle based on maximum-likelihood detector, IET Image Process. 7 (5) (2013) 451–463.

[39] Zou Ke, Chen Zhihao, Yuan Xuedong, Shen Xiaojing, Wang Meng, Fu Huazhu, A review of uncertainty estimation and its application in medical imaging, Meta Radiol. 1 (1) (2023) 100003.

[40] Rani Ritu, Jaiswal Garima, Nancy Lipika, Bhushan Shashi, Ullah Fasee, Singh Prabhishek, Diwakar Manoj, Enhancing liver disease diagnosis with hybrid SMOTE-ENN balanced machine learning models—an empirical analysis of Indian patient liver disease datasets, Front. Med 12 (2025) 1502749. 10.3389/fmed.2025.1502749 PMC12149218 40495970

[41] Mishra Amit Kumar, Singh Jagendra, Gupta Anurag, Singh Manoj Kumar, Singh Prabhishek, Diwakar Manoj, Deep learning-based diagnostic system for liver tumor classification using dynamic contrast-enhanced MRI images combined with clinical data, in: 2024 International Conference on Advances in Computing, Communication and Materials, ICACCM, IEEE, 2024, pp. 1–6.

[42] Simpson Amber L, Peoples Jacob, Creasy John M, Fichtinger Gabor, Gangai Natalie, Keshavamurthy Krishna N, Lasso Andras, Shia Jinru, D’Angelica Michael I, Do Richard KG, Preoperative CT and survival data for patients undergoing resection of colorectal liver metastases, Sci. Data 11 (1) (2024) 172. 38321027 10.1038/s41597-024-02981-2 PMC10847495

[43] Fedorov Andriy, Beichel Reinhard, Kalpathy-Cramer Jayashree, Finet Julien, Fillion-Robin Jean-Christophe, Pujol Sonia, Bauer Christian, Jennings Dominique, Fennessy Fiona, Sonka Milan, , 3D slicer as an image computing platform for the quantitative imaging network, Magn. Reson. Imaging 30 (9) (2012) 1323–1341. 22770690 10.1016/j.mri.2012.05.001 PMC3466397

[44] Isensee Fabian, Wald Tobias, Ulrich Constantin, Baumgartner Michael, Roy Saikat, Maier-Hein Klaus, Jaeger Paul, Nnu-net revisited: A call for rigorous validation in 3D medical image segmentation, 2024, arXiv preprint, URL https://arxiv.org/abs/2404.09556.

[45] Jäger Paul F, Lüth Carsten, Klein Lukas, Bungert Till, A call to reflect on evaluation practices for failure detection in image classification, in: ICLR 2023, 2023.

[46] Isensee Fabian, Wald Tassilo, Ulrich Constantin, Baumgartner Michael, Roy Saikat, Klaus Maier-Hein, Jaeger Paul F, Nnu-net revisited: A call for rigorous validation in 3d medical image segmentation, in: International Conference on Medical Image Computing and Computer-Assisted Intervention, Springer, 2024, pp. 488–498.

